# COX7A2L/SCAFI and Pre-Complex III Modify Respiratory Chain Supercomplex Formation in Different Mouse Strains with a *Bcs1l* Mutation

**DOI:** 10.1371/journal.pone.0168774

**Published:** 2016-12-20

**Authors:** Mina Davoudi, Heike Kotarsky, Eva Hansson, Jukka Kallijärvi, Vineta Fellman

**Affiliations:** 1 Pediatrics, Department of Clinical Sciences, Lund, Lund University, Lund, Sweden; 2 Folkhälsan Research Center, Helsinki, Finland; 3 Children’s Hospital, University of Helsinki, Helsinki, Finland; Virginia Commonwealth University, UNITED STATES

## Abstract

The COX7A2L (Supercomplex Assembly Factor I, SCAFI) protein has been proposed to be a mitochondrial supercomplex assembly factor required for respirasome (supercomplex containing complexes I, III, and IV) formation. In the C57BL/6 mouse strain a homozygous in-frame 6-base-pair deletion in the *COX7a2l/SCAF1* gene resulting in unstable protein and suggesting loss of function was previously identified. The loss of SCAFI was shown to impede respirasome formation, a major concern for the use of C57BL mouse strains in mitochondrial research. In contradiction, another recent study suggested that supercomplex formation is independent of SCAFI isoforms. We investigated whether SCAFI isoform status affected the disease severity and supercomplex formation in the liver of *Bcs1l*^*c*.*232A>G*^ knock-in mice with incomplete complex III assembly. In homozygotes (*Bcs1l*^*G/G*^) of mixed (C57BL/6:129/Sv) genetic background, the lifespan was similar in mice with wild-type *SCAFI* allele and in those homozygous (*SCAFI*^short/short^) for the deleted SCAF1 variant (34±3 days; n = 6 vs. 32±2 days; n = 7, respectively). SCAFI heterozygosity (*SCAFI*^long/short^) resulted in decreased SCAFI protein but respirasome assembly was unaffected. Congenic (C57BL/6) mice were of the genotype *SCAFI*^short/short^ and had no detectable SCAFI protein. In their liver mitochondria, respirasome composition was altered as compared to mixed background mice. Complex IV was mainly present as monomers and dimers, and only low amounts were found in combination with complex I and complex III or with precomplex III. The main supercomplex in the liver mitochondria of C57BL/6 mice comprised only complexes I and III. In conclusion, in liver mitochondria of C57BL/6 mice, supercomplexes had markedly reduced amount of, but were not completely depleted of, complex IV, supporting a role for COX7A2L/SCAFI in supercomplex assembly. However, the disease progression of the *Bcs1l* mutant mice was unrelated to SCAFI isoforms and supercomplex composition, suggesting that other genetic factors contribute to the different survival in the different genetic backgrounds.

## Introduction

Mitochondrial respiratory chain (RC) consists of four complexes (CI-CIV), which use the energy of electron transport to generate a proton gradient that drives ATP synthesis by the ATP synthase (complex V, CV). The presence of larger conglomerates (supercomplexes) of the RC complexes was first suggested in 2000 [1] and has been supported by several other studies. The RC is considered a dynamic apparatus composed of both individual complexes and their association into supercomplexes, which usually are combinations of CI together with CIII or with both CIII and CIV (the so called respirasome) or CIII with CIV [2]. Supercomplex formation requires the presence of cardiolipin [3, 4] and specific assembly factors that have been identified both in yeast [5–7] and mammals [8, 9]. Supercomplexes are enzymatically active [2] and influence the efficacy of the RC [9].

COX7A2L, also named SCAFI (Supercomplex Assembly Factor I) has been suggested to be indispensable for the inclusion of CIV in supercomplexes [9]. Two variants of the *SCAFI* gene and protein were found in commonly used laboratory mouse strains. In C57BL/6 mice, the gene harbors a six-base-pair in-frame region deletion encoding a shorter (111 amino acids), unstable protein, whereas full length SCAFI protein (113 amino acids) is present in the 129/Sv strain having the wild-type *SCAFI* gene [9]. In comparison to respirasomes containing CIV, respiration in liver mitochondria lacking CIV in supercomplexes due to the deletion in SCAFI was slightly higher with substrate for only CI (pyruvate and malate) or CII (succinate), but there was no difference when both substrates were combined. The study suggested that supercomplex formation might have important effects on the phenotype of mouse disease models [9]. A contradictory study found that CIV was present in supercomplexes and RC function was normal in mitochondria of wild-type C57BL/6 mice with short *SCAFI* alleles [10]. It is not known whether the short/short genotype has an effect on the phenotype and disease progress in mouse models of RC complex deficiencies.

We set out to investigate if the SCAFI variants modify disease severity and supercomplex formation in liver mitochondria in mice with mitochondrial hepatopathy due to CIII deficiency. Our knock-in mouse model harbors the same point mutation (*Bcs1l* c.232A>G, p.S78G) that in humans causes the GRACILE syndrome, a neonatal lethal mitochondrial disorder presenting with fetal-onset growth restriction and hepatopathy [10]. The homozygous mutation results in decreased incorporation of Rieske iron-sulfur protein into CIII and thereby in CIII deficiency in both humans and mice [11, 12]. The main supercomplex in liver mitochondria of these homozygous mice contains CI, precomplex III without Rieske iron-sulfur protein and a small amount of fully assembled CIII [13]. The majority (95%) of mutant mice of mixed (129/Sv:C57BL/6) genetic background develop symptoms before postnatal day 40 (P40), while a minority (5%) develop no other symptoms than growth restriction until P70-165 [12]. In congenic mice backcrossed to the C57BL/6 strain, however, the disease progression is more rapid resulting in lethality before P30. We previously reported that, in liver mitochondria of the mutant mice of the C57BL/6 background, CIV was not conclusively found in supercomplexes [13]. This suggests that the strain carries the deleted SCAFI variant, which may cause loss of CIV from supercomplexes. We hypothesize that the difference in survival of the *Bcs1l* mutant mice between the two different genetic backgrounds might be ascribed to differences in supercomplex formation and thus respiration efficiency.

## Material and Methods

### Animal experiments

Mixed background (129/Sv:C57BL/6) mice harboring the *Bcs1l*^c.232A>G^ mutation [12] were backcrossed for 10–12 generations to achieve mutant mice in congenic C57BL/6 (substrain C57BL/6NCrlLtcf) background [13]. Sick homozygous (*Bcs1l*^*G/G*^) mice and their littermate wild-type (*Bcs1l*^*A/A*^) or heterozygous (*Bcs1l*^*A/G*^) control animals of both strains were compared with 129/Sv wild-type mice. The animals were maintained on rodent diet (Labfor R34, Lactamin, Stockholm, Sweden) and water ad libitum in a vivarium with 12 h light/dark cycle at 22°C. After weaning at 21 days of age, the animals were observed daily and weight was monitored 2–5 times a week. A health score was developed based on six typical behavioral characteristics: waddling gait, reduced curiosity, lack of movement in the cage, appearance of kyphosis, deterioration of balance and loss of grip strength. Each item was scored from zero to two (0 for normal, 1 for slight abnormality and 2 for clear abnormality). When weight gain was decreased compared to the matched littermate control wild-type (or heterozygous) mouse, scoring was performed in connection with the weighing procedure. When the score was ≥7/12 or no weight gain occurred, the animals were considered as having end-stage disease and were sacrificed to prevent spontaneous death. No unexpected deaths occurred in the litters studied. The researchers were unaware of the SCAFI genotype during the entire lifespan. *Bcs1l*^*G/G*^ mice of mixed genetic background homozygous for the short SCAFI allele were chosen from different litters as those with the long allele to exclude risk of selection bias for an earlier sacrificing time point for the second (or third) *Bcs1l*^*G/G*^ animal in the litter. The animals were sacrificed by cervical dislocation without premedication to minimize handling. Liver tissue samples were collected immediately after sacrificing for histology, mitochondrial isolation and snap-freezing.

### Ethics statement

Animal experiments were performed according to national guidelines with the approval of the Lund regional animal research ethics committee (permissions M253-08, M245-11). All efforts were taken to ameliorate suffering.

### Genotyping for supercomplex assembly factor I (*SCAFI*)

DNA isolated from tail biopsies was used for detection of *SCAFI* variants (long and short) by polymerase chain reaction (PCR), using primers P1 (5´- CTTTCTTGCTTTGCAGAAGGC-3´) and P2 (5´GAAGGCCTCGTTTCAGGTGG-3´) [9] and *Taq* polymerase from Fermentas GmbH, St. Leon-Rot, Germany. The PCR products were analyzed on 12% polyacrylamide gels.

### Isolation of mouse liver mitochondria and purification of complexes

Mouse liver samples were obtained from the three animal strains (129/Sv:C57BL/6, congenic C57BL/6NCrlLtcf and wild-type 129/Sv). Mitochondria were isolated from tissue homogenates by sequential centrifugation including density purification on 19% Percoll (GE Healthcare, Amersham, UK) as previously described [12]. Isolated mitochondria were stored at -80°C. They were used for CIII activity measurements with ubiquinol as electron donor in a spectrophotometric assay [12]. Mitochondrial complexes and supercomplexes were extracted from isolated mitochondria by incubation with 0.8% digitonin (Sigma Aldrich, Stockholm, Sweden) as previously described [13].

### SDS-PAGE, BNGE and 2D-BNGE

For detection of SCAFI protein, liver tissue lysates were run on a 12% SDS-PAGE. RC complexes and supercomplexes were assessed with blue native gel electrophoresis (BNGE) or 2-dimensional BNGE (2D-BNGE) as described earlier [13]. Proteins separated by SDS-PAGE, BNGE or 2D-BNGE were blotted onto polyvinylidine difluoride membranes using iBlotTM equipment (Invitrogen, Carlsbad, CA, USA). Membranes were blocked in PBS supplemented with 0.05% Tween 20 and 5% dry milk for subsequent antibody incubation.

The SCAFI protein was detected by an antibody against COX7A2L (66062-1-lg, Proteintech Group, USA). Most antibodies directed against respiratory chain complex subunits were obtained from MitoSciences (Eugene, Oregon, USA); for CI subunit NDUFA9 (MS111) was used, for CIII subunits Core1 (MS303) and RISP (MS305), for CIV subunit I (COX1, MS404) and subunit Va (MS409). An antibody directed against ETFAα (MS782) was used as loading control. An antibody detecting the CI subunit NDUFV1, which is incorporated in the complex at the final assembly stage, was obtained from Sigma Aldrich (Stockholm, Sweden). Primary antibodies were detected by incubation with HRP-coupled goat anti-mouse secondary antibody (DAKO Cytomation, P0447). Membranes were developed with ECL plus (GE Healthcare, Amersham, UK). For detection of COX1 a prolonged exposure time was used (up to 10 min). The 2D-BNGE membranes were first probed with RISP and COX1 antibodies, thereafter stripped with Re-blot Plus antibody stripping solution (Millipore, Temecula, CA, USA), then probed with antibody against Core1 and after a second stripping probed with antibody against NDUFA9.

### Statistics

The group differences in lifespan were analyzed with Student´s t-test. A *p* value below 0.05 was considered statistically significant. The values are presented as mean and SD. Differences in 2-D-BNGE immunoblots were calculated with densitometry.

## Results

### Disease progression in homozygotes *(Bcs1l*^*G/G*^*)* of different genetic backgrounds is not related to SCAFI isoforms

**Fig 1:**
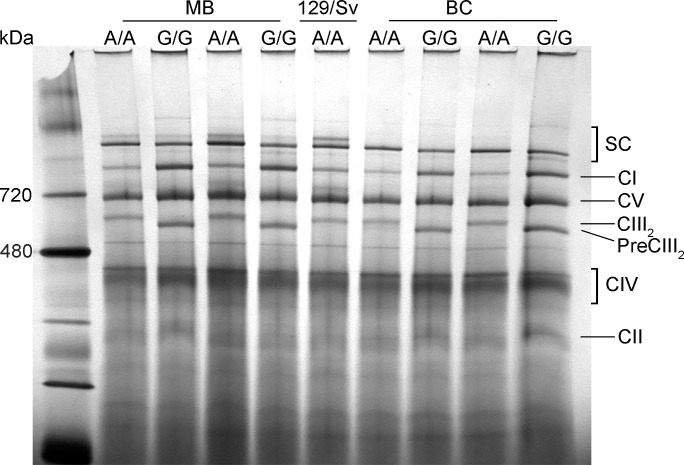
BNGE analysis. Comparison of isolated liver mitochondria from *Bcs1l* mutant homozygotes (G/G) and wild-types (A/A) of 129/Sv:C57BL/6 mixed background (MB), C57BL/6 backcrossed (BC) strain and the 129/Sv strain. Four supercomplex bands (SC) are visible in wild-type animals in MB and 129/Sv strains, one of the bands not found in C57BL/6 animals. In homozygotes (G/G) in both MB and BC strains, free complex I (CI) is more abundant than in wild-type animals (A/A), and free complex III (CIII) is only present as a pre-complex (PreCIII_2_).

Health status in homozygous mutant (*Bcs1lG/G*) and wild-type (*Bcs1lA/A*) littermate control mice of mixed background (129/Sv:C57BL/6) [12] was compared with *Bcs1lG/G* and *Bcs1lA/A* of backcrossed C57BL/6 background [13] and with 129/Sv wild-type mice. The homozygous mutant mice in both genetic backgrounds developed normally until P21, where after their growth slowed down [12, 13]. Because of imminent lethality and decreasing weight the backcrossed *Bcs1lG/G* mice were sacrificed by P30, whereas those of mixed background were euthanized by about P40. Histopathological analyses of the liver of backcrossed C57BL/6 *Bcs1lG/G* mice displayed early-stage hepatopathy and centrilobular glycogen depletion (S1 Fig) somewhat more severe than in the mixed background strain [12]. In BNGE of isolated mitochondria from liver of homozygotes, CIII was typically present as a pre-complex [13] because of the *Bcs1l* mutation (Fig 1).

**Fig 2:**
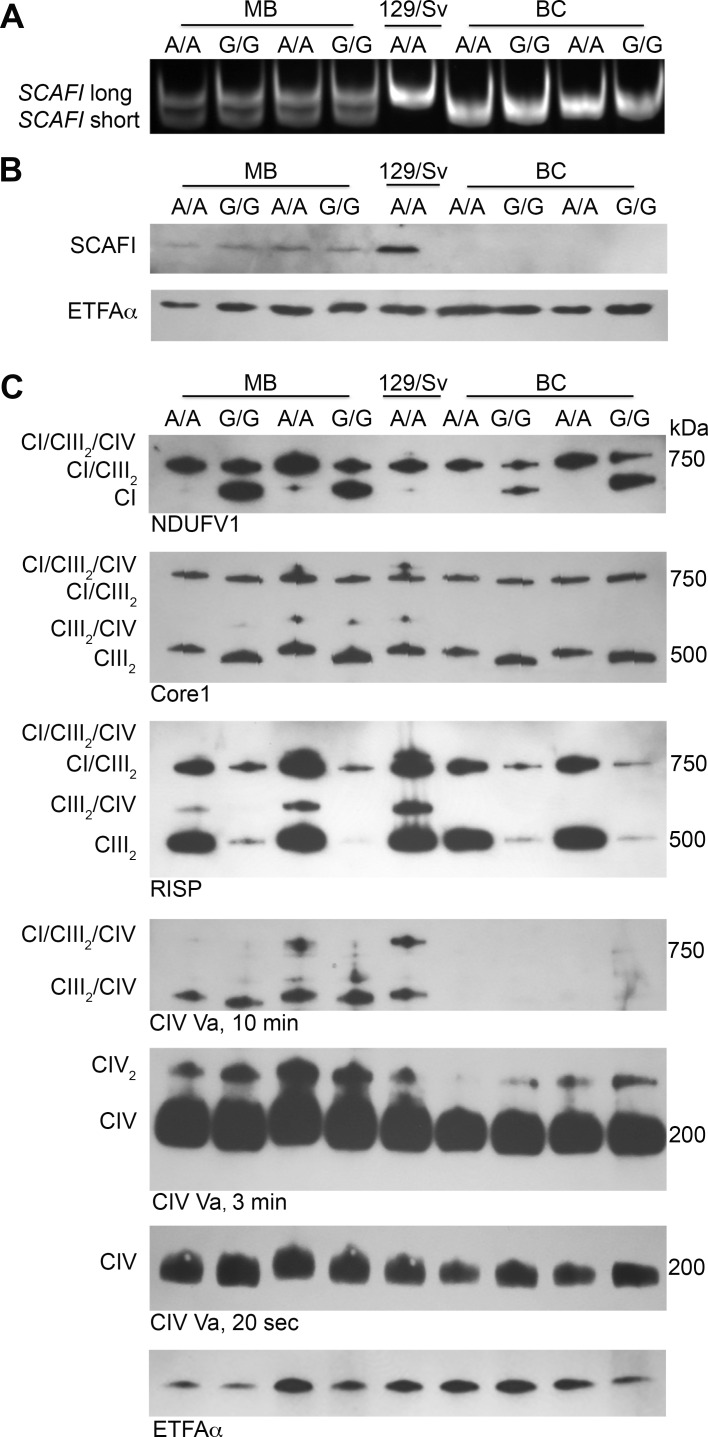
*SCAFI* allele status and its effect on supercomplex assembly in *Bcs1l* mutant mice (G/G) of different genetic backgrounds. Wild-types (A/A) and *Bcs1l* mutant mice (G/G) of 129/Sv:C57BL/6 mixed background (MB) and C57BL/6 background (BC) were analyzed and a wild-type of the 129/Sv strain (129/Sv) was used as a control. (A) Agarose gel electrophoresis of SCAFI genotyping PCR products showing the long (*SCAFI* long) and short (*SCAFI* short) alleles in mice of different genetic backgrounds. (B) Western blot analysis of SCAFI protein and ETFAα (electron transfer flavoprotein alpha) as loading control in liver tissue lysate showing reduced level of SCAFI protein in mixed background (MB) mice and no detectable protein in C57BL/6 mice (BC). (C) Respiratory chain complexes and supercomplexes were separated on BNGE and detected by Western blot using the antibodies indicated. Molecular weights are indicated on the right. Note that the COX1 antibody required an extended exposure time for detection in supercomplexes. ETFAα was used as a loading control. NDUFV1 antibody shows in mutant (G/G) mice abundant free CI, in all mice CI/CIII_2,_ and in wild-type MB and 129/Sv that this is overlapping with the band for CI/CIII_2_/CIV. Core1 shows pre-CIII_2_ and CIII_2_, and RISP fully assembled CIII_2_ indicating presence of CIII in all supercomplexes of 129/Sv and wild-type MB animals, but mainly in supercomplex CI/CIII_2_ in mutant (G/G) mice of both backgrounds. CIV antibody shows a clear band in respirasome in 129/Sv mouse, but of varying intensity in MB wild-type mice in relation to the loading control (ETFAα).

*SCAFI* genotyping showed that 129/Sv wild-type mice were homozygous for the long allele encoding a 113-amino acid protein, whereas mice of the C57BL/6 backcrossed strain were homozygous for the short allele predicting a 111-amino acid protein that was undetectable on Western blots (Fig 2A and 2B). Mice of mixed background were either heterozygous for the two alleles (Fig 2A) or homozygous for the short one. The 113 amino acid SCAFI protein was detected in 129/Sv wild-type (homozygous for long *SCAFI*, Fig 2B). The backcrossed C57BL/6 strain lacked detectable SCAFI protein, whereas the 129/Sv:C57BL/6 strain heterozygous for long *SCAFI* had detectable SCAFI protein but lower expression than in 129/Sv wild-type mice.

The typical 1-month lifespan of the homozygotes in mixed background was not related to SCAFI isoform status (Table 1). Exceptionally long survivors were one homozygote with *SCAFI*^long/short^ (105 days, weight 13.1 g, 47% of littermate control) and two with *SCAFI*^short/ short^ (60 days, 10.6 g, 54% and 170 days 22.2 g, 59%), respectively. The CIII activity was similar in wild-type animals of both strains and the decrease in activity in the mutant mice was also similar at end-stage disease in both genetic backgrounds (activity level 10–20% of littermate controls, data in Supplement S1 Table).

**Table 1:** *SCAFI* genotype and age at end-stage disease (age at sacrificing) of homozygous (*Bcs1l*^*G/G*^) mice in different genetic backgrounds.

Mouse strain of *Bcs1l*^*G/G*^	*SCAFI* genotype	Age at sacrificing (days)	
		Typical age^a^	Long survival
129/Sv:C57BL/6	long/short	34 ± 3 (n = 6^b^)	105 (n = 1)
129/Sv:C57BL/6	short/short	32 ± 2 (n = 7^c^)	60; 170 (n = 2)
C57BL/6	short/short	28 ± 1^d^ (n = 8)	None

^a^Values are mean ± SD

^b^Includes one mouse with long/long genotype

^c^The mice with short/short genotype are from different litters than those with long/short

^d^The lifespan of C57BL/6 homozygotes was significantly (calculated with t-test) shorter than that of 129/Sv:C57BL/6 mixed genetic background with long/short (*p*<0.001) and short/short (*p* = 0.003) *SCAFI*.

### Long *SCAFI* isoform is required for normal CIV assembly into supercomplexes

**Fig 3:**
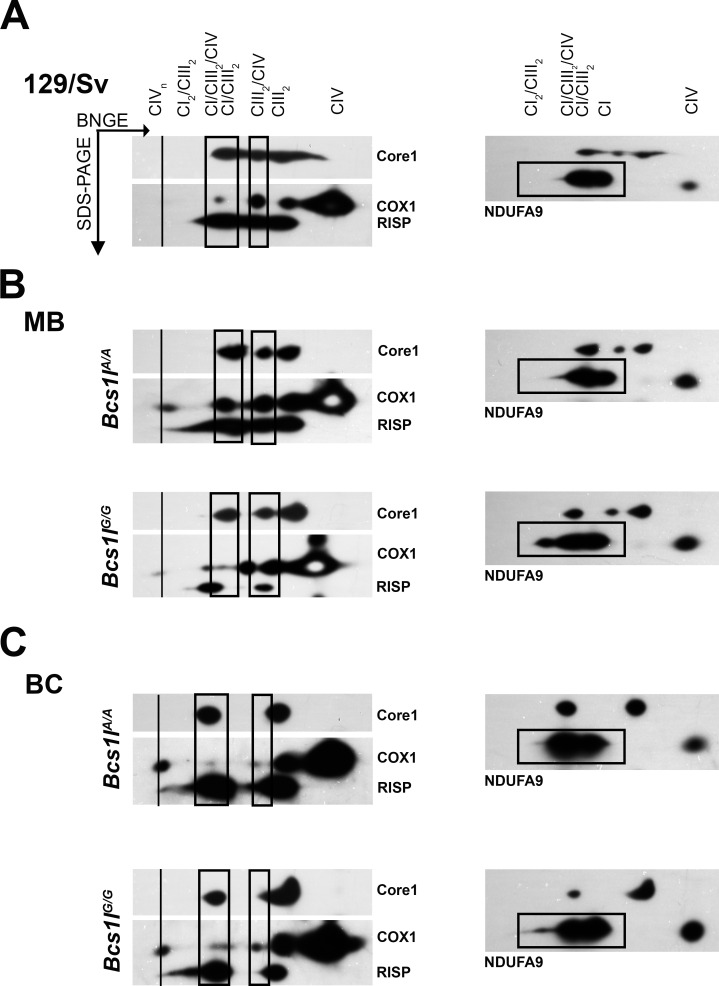
2D-BNGE with Western blot detection showing supercomplex composition in mitochondria of the three mouse strains studied. Antibodies against CIII (RISP) and CIV (COX1) subunits were initially used and after stripping the antibody against Core1. After a second stripping the antibody against CI (NDUFA9) was used. Supercomplexes are indicated by vertical boxes. (A) In the 129/Sv wild-type mouse, CIV was present in two supercomplexes. (B) Both CIV containing supercomplexes were present in wild-type mice (*Bcs1l*^*A/A*^) of 129/Sv:C57BL/6 mixed background strain (MB with *SCAFI*^*long/short*^). In mutant (*Bcs1l*^*G/G*^) mice, CIV was decreased in the respirasome CI/CIII_2_/CIV and more abundant in the formation of pre-CIII_2_/CIV. (C) In backcrossed (BC) C57BL/6 congenic strain (with *SCAFI*^*short/short*^), CIV was very faintly present in supercomplexes even when exposure time was increased compared to MB. In homozygotes (G/G) of both backgrounds, a CI_2_/CIII_2_ supercomplex was found and free CI was more abundant than in wild-type (assessed with densitometry: 26% in 129/Sv, 27% vs. 49% in MB and 48% vs. 56% in BC wild-type and homozygotes, respectively), as also shown in Fig 2.

In BNGE of liver mitochondria, four different supercomplexes were identified in mixed background and 129/Sv wild-type mice (Fig 1). Both in BNGE (Fig 2C) and 2D-BNGE (Fig 3) analyses, the most prominent supercomplex contained CI/CIII_2_, detected by antibodies against subunits NDUFV1 and Core1 (Fig 2C). The antibody against subunit Va detected CIV mainly as free monomer and dimers (Fig 2C). In BNGE of mitochondria from 129/Sv mice (*SCAFI*^*long/long*^) and mixed background wild-type mice (*SCAFI*^*long/short*^), CIV was part of supercomplexes CIII_2_/CIV and CI/CIII_2_/CIV, whereas in the C57BL/6 background (*SCAFI*^*short/short*^) these supercomplexes were not detected with the antibodies used (Fig 2C). In mixed background homozygous (*Bcs1lG/G*) mice, pre-complex III_2_ containing Core1, but not Rieske subunit and thus of a smaller molecular weight than CIII, was found together with CIV in CIII_2_/CIV supercomplex (Fig 2C).

As we did not convincingly find CIV in supercomplexes in backcrossed animals by BNGE, we performed 2D-BNGE runs to separate subunits of the complexes and investigate the presence of CIV. CI and CIII were abundant in supercomplexes in all three strains, whereas CIV was mainly present in free form as monomers and dimers (Fig 3). In the 129/Sv wild-type mice, CIV was detected both together with CIII (CIII_2_/CIV) and in the CI/CIII_2_/CIV supercomplexes. In wild-type mice of mixed background (*SCAFI*^*long/short*^), CIV was also present in CIII_2_/CIV and CI/CIII_2_/CIV supercomplexes (Fig 3B), but in *Bcs1lG/G* homozygotes diminished amounts of CIV were detected in the CI/CIII_2_/CIV supercomplex. In CI/CIII_2_/CIV the RISP antibody revealed a strong band but not in CIII2/CIV (as shown by Core1 antibody) suggesting that pre-CIII and CIV are associated in the CIII2/CIV supercomplex in these homozygotes (Fig 3B). With a longer exposure time the COX1 antibody detected a faint band in CI/CIII2/CIV area both in congenic wild-type (*Bcs1lA/A*) and homozygous (*Bcs1lG/G*) mice (Fig 3C). Further, a similar faint COX1 band was detected for CIII2/CIV, but it was not co-localized with the Rieske subunit in the *Bcs1lG/G* homozygous mouse (Fig 3C, S2 Fig), suggesting that this supercomplex contained pre-complex III like in homozygotes of mixed background (Fig 3B).

Mitochondria from *Bcs1lG/G* mice of both mixed and congenic background displayed an additional supercomplex detected with RISP and NDUFA9 larger than CI/CIII_2_/CIV.

The uncropped blots of the membranes probed with Core1 antibody after the first stripping to remove RISP and COX1 antibodies show the alignment of Core1 and RISP, and that COX1 antibody reactivity has been removed from the area positive for NDUFA9 antibody (S2 Fig).

## Discussion

Our hypothesis that the longer survival of homozygous *Bcs1l* mutant mice in mixed background would be related to presence of long SCAFI isoform was not supported. Previously, we have shown that respiration as assessed using Oroboros Oxygraph is similar in homozygotes of C57BL/6 strain [13] and in those of mixed background [12], both when using substrates for CI and CII. Thus, we have not found any functional differences in RC between mutants in the different genetic backgrounds. SCAFI may not be crucial for mitochondrial function since knockout of the *SCAFI* gene (*Cox7rp)* resulted in a mild phenotype with decreased COX activity and ATP production in muscle, but the mice were otherwise healthy and fertile [8].

Supercomplex formation has been suggested to modify respiratory chain activity in response to the metabolic state and energy need of the organism [9]. Thus, increased metabolic pressure such as that in the *Bcs1lG/G* mice with progressive CIII deficiency might be compensated by increased supercomplex assembly. In line with data from mouse liver mitochondria showing that SCAFI is needed for CIV incorporation into supercomplexes [9], we found abundant CIV in the combinations CIII2/CIV and CI/CIII2/CIV only in mice of mixed background with the long *SCAFI* allele, but very little in mice homozygous for the short allele. Ikeda et al. have shown that loss of function of *Cox7rp* (synonym for *Cox7a2l* and *SCAFI*) in the C57BL/6 background affected mainly the CI/CIII2/CIV supercomplex formation, whereas the CIII2/CIV supercomplex was still detectable in muscle mitochondria [8]. These differences might be attributed to tissue-specific differences between liver and muscle mitochondria. Using radiolabelled precursor of the COX6a subunit of CIV, the pattern of supercomplexes was found to be similar in C57BL/6J (homozygous for the short *SCAFI* allele) and control liver mitochondria (with long *SCAFI* allele), but the amount of CIV was clearly less in supercomplexes in C57BL/6J mitochondria [10]. Our BNGE conditions have been carefully titrated and were similar to theirs in the experiments presented here. Thus, we consider our findings to be representative for liver mitochondria of the C57BL/6 mouse strain. It remains to be elucidated whether other interacting factors modify the effect of SCAFI on supercomplex formation. In *Bcs1l* mutant mice, the accumulation of pre-CIII in mitochondria of both genetic backgrounds, as a result of the progressive Rieske protein depletion [13], influenced supercomplex formation. The small amount of fully assembled CIII2 was associated with the CI/CIII2/CIV respirasome suggesting a preferred rearrangement towards CI/CIII2 if not fully assembled. Supercomplexes have been reported to be sensitive to reactive oxygen species (ROS) [14]. Disruption of the supercomplex CI/CIII2 results in enhanced ROS production from CI [15]. Thus, the supercomplex rearrangement might prevent generation of ROS from CI and could explain why elevated ROS levels were detectable only in end-stage disease in *Bcs1l*^*G/G*^ mice of mixed genetic background [16].

In conclusion, we show that COX7a2L/SCAFI is important for formation of respiratory chain supercomplexes containing CIV. However, in mice with short *SCAFI* isoforms, a small amount of CIV was still detectable in supercomplexes. Whether this formation is the result of only partial loss of function of SCAFI by the 6-base-pair deletion (hypomorphic allele) or to other factors contributing to the assembly remains to be resolved. In a recent transomics study on tens of wild-type mouse strains, the COX7a2L/SCAFI protein was found to be involved in the formation of many CIV containing supercomplexes, but the influence varied between tissues [17]. The presence of pre-CIII in our homozygous mutant mice with defective Rieske iron sulfur protein incorporation influenced also supercomplex configuration. In our viable mitochondrial disease model, the loss of CIV in the respirasome-supercomplex did not have a detectable functional effect, in contrast to the previous study on wild-type animals [9]. As for the functional consequences of SCAFI loss-of-function our results support the findings of the Larsson group [10] and of the Inoue group [8]. Thus, although SCAFI clearly affects supercomplex assembly, it does not seem to be crucial for RC function in the liver mitochondria of *Bcs1l* mutant mice.

## Supporting Information

S1 Fig Liver histology. Hematoxylin-eosin (HE) staining showed normal liver architecture in control (A/G) mice (A, C) and localized hepatocyte hypertrophy with more eosinophilic appearance due to glycogen depletion in the mutant (G/G) livers of mixed 129/Sv:C57BL/6 background at P34 (B). In the mutant livers (G/G) of backcrossed C57BL/6 background, signs of early-stage hepatopathy with expansion of portal areas (densely packed blue nuclei) and incipient fibrosis were observed at P29. Increased number of cells with double nuclei, and scattered strongly eosinophilic (darker red) and degenerating (pale ballooned cytoplasm) hepatocytes were present (D). Periodic acid-Shiff (PAS) staining showed normal non-fasted glycogen (purple staining) distribution in control (A/G) mice of both backgrounds (E, G) and typical patchy glycogen depletion in the mutant mice (G/G, F, H) with more prominent periportal depletion in the backcrossed C57BL/6 mice (H). Original magnification 200X.
(TIF)

S2 Fig Uncropped panels of 2D-BNGE. The membranes were initially probed with RISP and COX1 antibodies and after stripping probed with Core1 antibody. COX1 antibody was almost completely removed from SC. In BC animals the main supercomplex (SC) formation is with CI and CIII with small amount of CIV, containing both pre-CIII_2_ and fully assembled CIII_2_ in homozygous mutant mice (G/G).
(TIF)

S3 Fig Survival curves. Age at sacrificing of homozygotes (*Bcs1l*^*G/G*^) of mixed genetic backgrounds with long/short or short/short *SCAFI* alleles and of congenic C57BL/6 strain with short/short alleles.
(TIF)

S1 Table Data on animals included in the study. (PDF)
