# Supplementary figures and images for: COX7A2L/SCAFI and Pre-Complex III Modify Respiratory Chain Supercomplex Formation in Different Mouse Strains with a Bcs1l Mutation
Source: PLoS One. 2016 Dec 20;11(12):e0168774. doi: 10.1371/journal.pone.0168774 (PMC5173253; doi:10.1371/journal.pone.0168774)

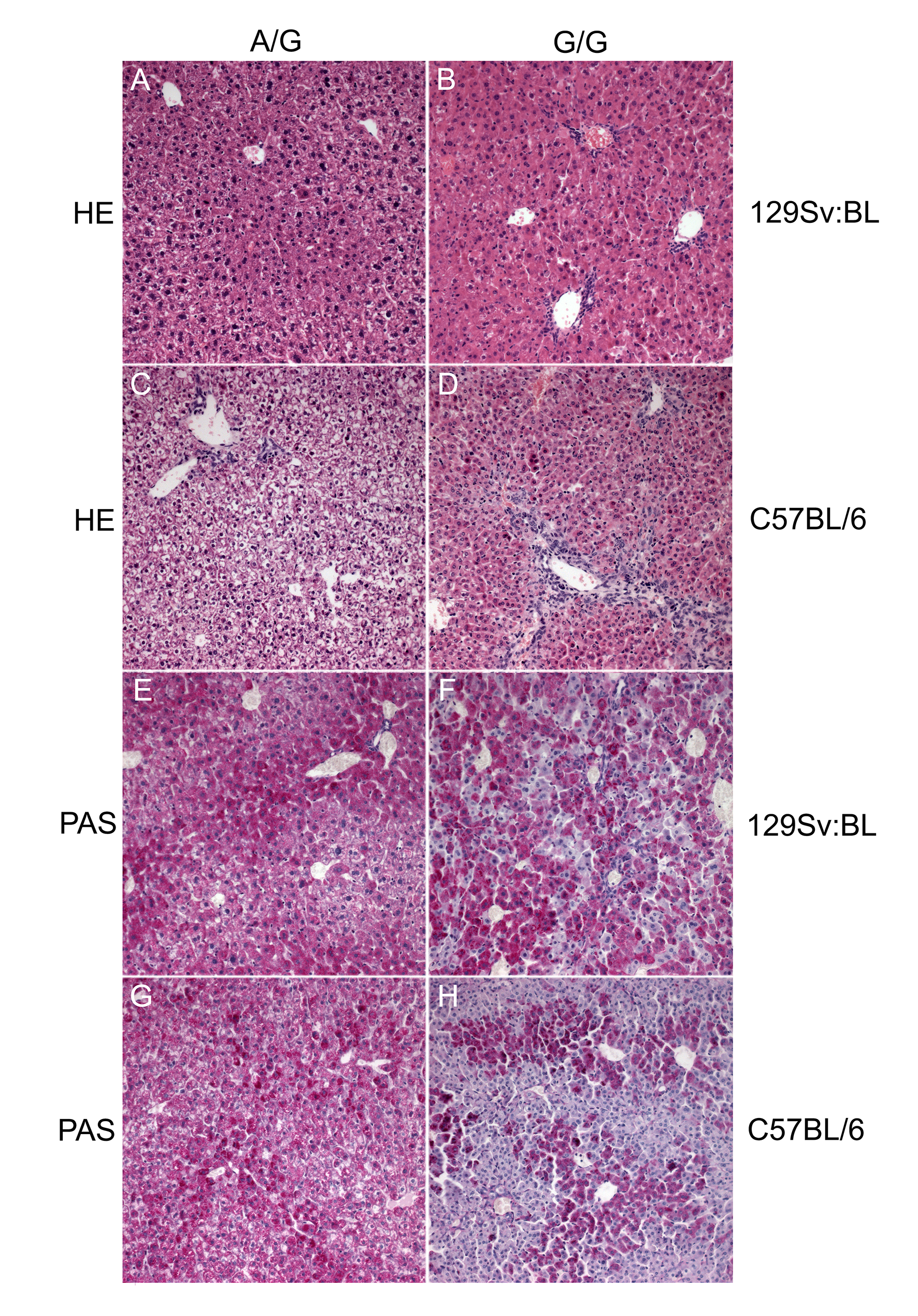

Supplement: S1 Fig — Hematoxylin-eosin (HE) staining showed normal liver architecture in control (A/G) mice (A, C) and localized hepatocyte hypertrophy with more eosinophilic appearance due to glycogen depletion in the mutant (G/G) livers of mixed 129/Sv:C57BL/6 background at P34 (B). In the mutant livers (G/G) of backcrossed C57BL/6 background, signs of early-stage hepatopathy with expansion of portal areas (densely packed blue nuclei) and incipient fibrosis were observed at P29. Increased number of cells with double nuclei, and scattered strongly eosinophilic (darker red) and degenerating (pale ballooned cytoplasm) hepatocytes were present (D). Periodic acid-Shiff (PAS) staining showed normal non-fasted glycogen (purple staining) distribution in control (A/G) mice of both backgrounds (E, G) and typical patchy glycogen depletion in the mutant mice (G/G, F, H) with more prominent periportal depletion in the backcrossed C57BL/6 mice (H). Original magnification 200X. (TIF) [file pone.0168774.s001.tif]

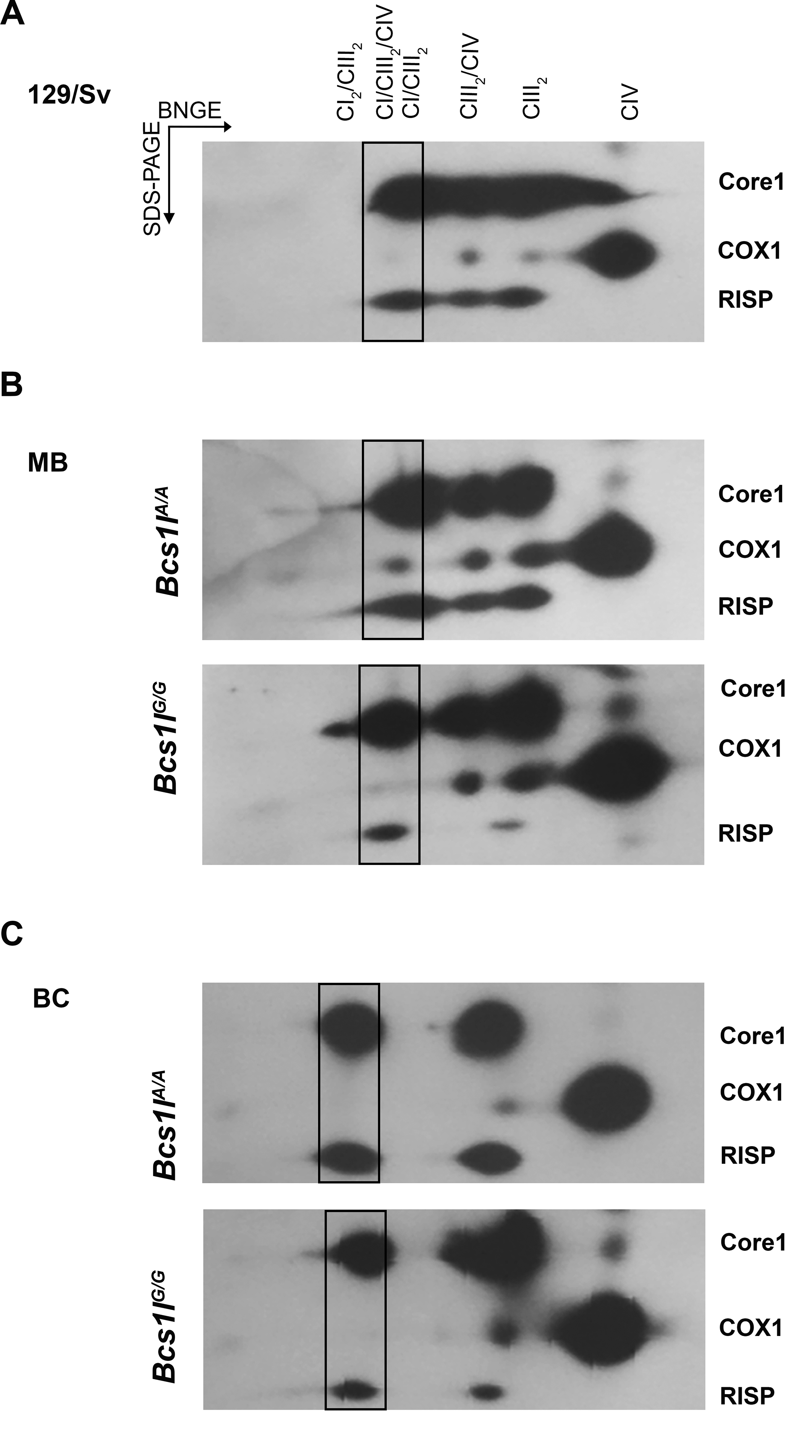

Supplement: S2 Fig — The membranes were initially probed with RISP and COX1 antibodies and after stripping probed with Core1 antibody. COX1 antibody was almost completely removed from SC. In BC animals the main supercomplex (SC) formation is with CI and CIII with small amount of CIV, containing both pre-CIII2 and fully assembled CIII2 in homozygous mutant mice (G/G). (TIF) [file pone.0168774.s002.tif]

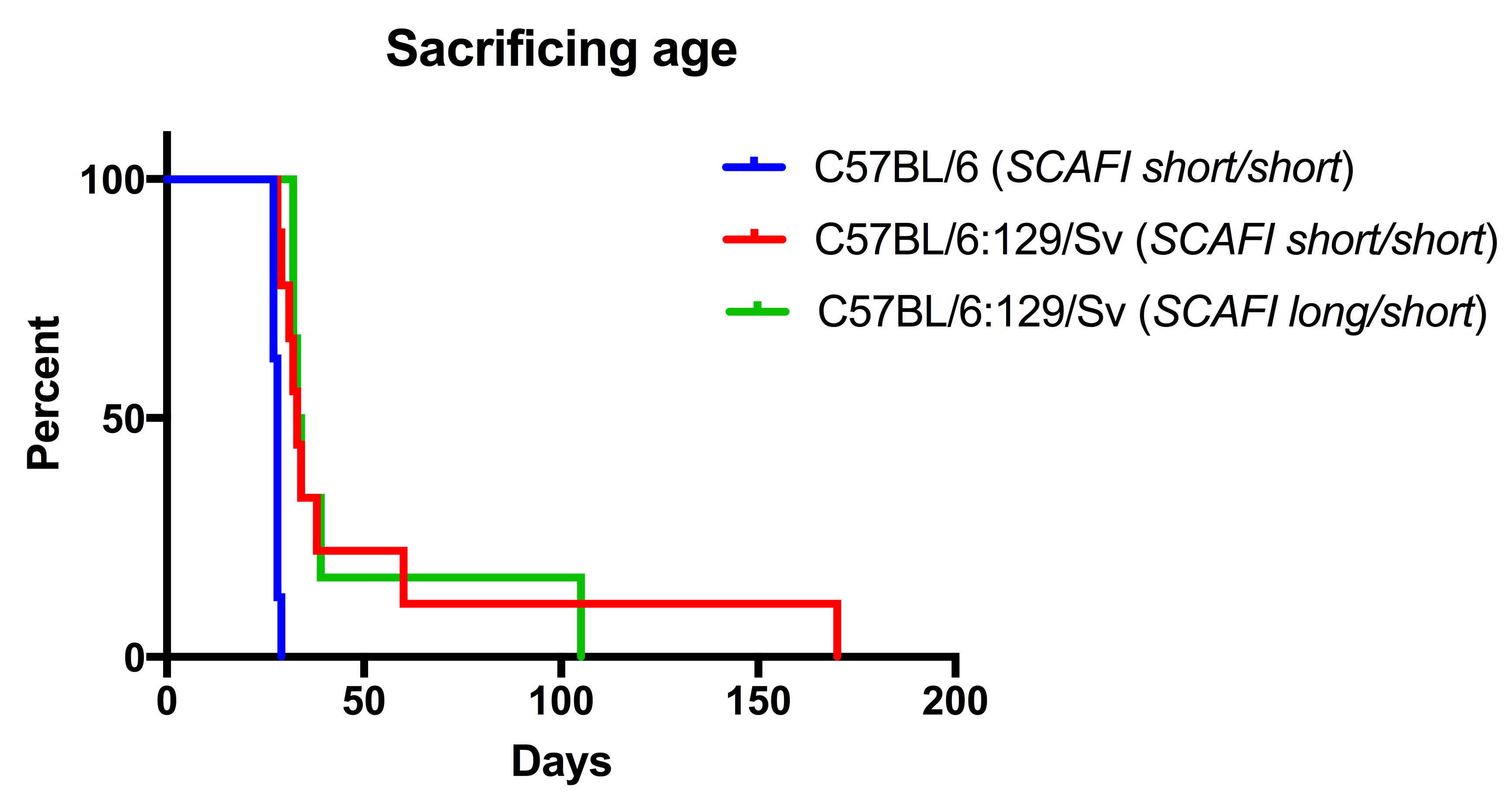

Supplement: S3 Fig — Age at sacrificing of homozygotes (Bcs1lG/G) of mixed genetic backgrounds with long/short or short/short SCAFI alleles and of congenic C57BL/6 strain with short/short alleles. (TIF) [file pone.0168774.s003.tif]
