# Supplementary material for: COX7A2L/SCAFI and Pre-Complex III Modify Respiratory Chain Supercomplex Formation in Different Mouse Strains with a Bcs1l Mutation
Source: PLoS One. 2016 Dec 20;11(12):e0168774. doi: 10.1371/journal.pone.0168774 (PMC5173253; doi:10.1371/journal.pone.0168774)
Supplement: S1 Table — (PDF) [file pone.0168774.s004.pdf]

S1 Table. Data on animals included in the study.

Each homozygote (G/G) was gender matched with a litter mate wild-type (A/A) or heterozygous (A/G) control mouse.
Heterozygotes are healthy with no differences compared to wild-type animals [12].
Gender:, M, male and F, female.
Age: the sacrificing age when health scoring or weight indicates end stage disease severity.
Background: MB, mixed background (129/Sv:C57BL/6) and BC, backcrossed (C57BL/6 substrain C57BL/6NCrlLtcf)

Study-ID	Genotype	Gender	Age, d	Background	PCR/SCAFI	CIII activity delta abs/30s	Weight, g
1	G/G	M	32	MB	long/short		
2	A/A	F	60	MB	long/long		19,8
3	G/G	F	60	MB	short/short		10
4	A/G	F	105	MB	long/short		28,1
5	G/G	F	105	MB	long/short		13,1
6	A/A	F	33	MB	long/long		
7	G/G	F	33	MB	long/long		
8	A/G	M	170	MB	long/short		
9	G/G	M	170	MB	short/short		22,2
10	A/G	F	28	MB	short/short		14,2
11	G/G	M	28	MB	short/short		10,6
12	A/A	F	29	MB	short/short		15,7
13	G/G	F	29	MB	short/short		7,7
14	A/A	F	31	MB	short/short		14,1
15	G/G	M	31	MB	short/short		7,1
16	A/G	M	32	MB	short/short		16,1
17	G/G	M	32	MB	short/short		6,8

18	A/A	F	32	MB	long/short		13,6
19	A/A	F	33	MB	long/short		
20	G/G	M	32	MB	long/short		9,6
21	A/A	M	33	MB	short/short		14,7
22	G/G	M	33	MB	short/short		8,7
23	A/A	M	33	MB	long/short	0.0198	16,4
24	G/G	M	33	MB	long/short	0.0042	11,2
25	G/G	F	34	MB	long/short		10,8
26	A/A	F	34	MB	long/short		20,6
27	A/A	F	34	MB	short/short		14,2
28	G/G	F	4	MB	short/short		8,2
29	A/A	F	38	MB	short/short		15,2
30	G/G	M	38	MB	short/short		9,6
31	G/G	F	39	MB	long/short	0.0026	11,4
32	A/G	M	39	MB	long/short	0.036	15,7
33	A/G	M	27	BC	short/short		
34	A/A	M	27	BC	short/short		17,4
35	G/G	F	27	BC	short/short		8,3
36	G/G	M	27	BC	short/short	0.0022	8,7
37	A/A	M	27	BC	short/short	0.0185	12,6
38	G/G	M	27	BC	short/short	0.0024	10
39	A/A	M	27	BC	short/short	0.0246	13,5
40	A/G	F	28	BC	short/short	0.0153	10,9
41	G/G	F	28	BC	short/short	0.0033	8,3
42	A/G	M	28	BC	short/short		11,3
43	G/G	M	28	BC	short/short		8,3
44	A/G	M	28	BC	short/short		11,6

45	G/G	M	28	BC	short/short		7,4
46	A/A	M	28	BC	short/short		11,6
47	G/G	M	28	BC	short/short		8,1
48	A/G	M	29	BC	short/short		11,8
49	G/G	M	29	BC	short/short		7,7
